# Assessment of a Flavone-Polysaccharide Based Prescription for Treating Duck Virus Hepatitis

**DOI:** 10.1371/journal.pone.0146046

**Published:** 2016-01-05

**Authors:** Hongxu Du, Shuaibing Zhang, Meiyun Song, Yixuan Wang, Ling Zeng, Yun Chen, Wen Xiong, Jingjing Yang, Fangke Yao, Yi Wu, Deyun Wang, Yuanliang Hu, Jiaguo Liu

**Affiliations:** Institute of Traditional Chinese Veterinary Medicine, College of Veterinary Medicine, Nanjing Agricultural University, Nanjing, P R China; University of Florida, UNITED STATES

## Abstract

Because polysaccharide and flavone ingredients display good antiviral activity, we developed a flavone/polysaccharide-containing prescription that would be effective against duck viral hepatitis (DVH) and investigated its hepatoprotective effects. Flavones were derived from *Hypericum japonicum* (HJF) (entire herb of *Hypericum japonicum Thunb*) and *Salvia plebeia* (SPF) (entire herb of *Salvia plebeia R*. *Br*.), and polysaccharides were derived from *Radix Rehmanniae Recens* (RRRP) (dried root of *Rehmannia glutinosa Libosch*). This prescription combination was based on the theory of syndrome differentiation and treatment in traditional Chinese veterinary medicine. *In vitro* and *in vivo* experiments were conducted using the three single ingredients compared to the combined HRS prescription to determine their anti-duck hepatitis A viral (anti-DHAV) activity. The results showed that all experimental conditions displayed anti-DHAV activity, but the HRS prescription presented the best effect. To further investigate the hepatoprotective effect of the HRS prescription on DHAV-induced hepatic injury, we tested the mortality rate, the hepatic pathological severity score, plasma biochemical indexes of hepatic function, blood DHAV gene expression levels and peroxidation damage evaluation indexes and then analyzed correlations among these indexes. The results demonstrated that the HRS prescription significantly decreased the mortality rate, reduced the severity of hepatic injury, decreased the hepatic pathological severity score, depressed blood DHAV gene expression levels, and returned the indexes of hepatic function and peroxidation almost to a normal level. These results indicate that the HRS prescription confers an outstanding hepatoprotective effect, and we expect that it will be developed into a new candidate anti-DHAV drug.

## Introduction

Duck virus hepatitis (DVH) is an acute, rapidly spreading, highly contagious disease of young ducklings that is characterized by hepatitis. Hepatic injury is the only visible pathological manifestation of this disease. The pathogen was defined as duck hepatitis virus (DHV). DHV includes three distinct serotypes: DHV-1, DHV-2 and DHV-3 [1,2]. DHV-1 was recently classified as a member of the *Picornaviridae* family and renamed duck hepatitis A virus (DHAV) according to sequence analysis [3]. Among these serotypes, DHAV is considered to be the most harmful to the large-scale duck industry all over the world. Although an attenuated DHAV vaccine and a hyperimmune serum are effective, they cannot be widely used and other effective preventive and therapeutic medicines have not been developed. If clinical cases of this disease were to emerge, it would cause irreparable damage.

The fact that viral infections can induce a large number of free radicals has been confirmed by many studies, and the injuries caused by free radicals are considered to be one of the pathogenic mechanisms that is widely associated with RNA viruses [4,5]. Our previous research also demonstrated that a large amount of free radicals are generated during DHAV infection, and the mortality rate from this disease can be effectively improved by reducing free radical levels [6]. Free radicals are widely produced by living organisms and are highly reactive, leading to the peroxidation of lipids, proteins and DNA [7]. Excessive free radical production is one of the driving forces behind hepatic injury, and it plays a critical role in various liver diseases [8,9]. Studies on polysaccharides and flavonoids, main ingredients in traditional Chinese medicines (TCM), have suggested that both of them possess free radical scavenging abilities [10,11].

TCM has been used in multiple combinations of compounds in the form of processed natural products used to treat human and animal diseases by both Chinese and other Asian populations for thousands of years [12]. The most striking pathological change induced by DVH is hepatic injury, such as hepatorrhagia and hepatic congestion, and the main clinical symptoms are cramps, convulsions, opisthotonus and sudden death. According to the theory of syndrome differentiation and treatment in traditional Chinese veterinary medicine, DVH is considered to be a syndrome of wind-heat in the liver channel and of body fluids being consumed due to excessive blood heat induced by an exogenous evil source. It is considered proper to clear liver heat, to extinguish liver wind, to cool the blood and to remove toxic materials [13]. Therefore, we used *Hypericum japonicum*, *Radix Rehmanniae Recens* and *Salvia plebeian* to formulate a *Hypericum japonicum*-*Radix Rehmanniae Recens*-*Salvia plebeian* (HRS) prescription to treat DVH. *Hypericum japonicum* refers to the entire *Hypericum japonicum Thunb* herb, which is also named Diercao in China. The entire *Salvia plebeia R*. *Br*. (Lizhicao) herb is used in the TCM system. *Radix Rehmanniae Recens*, the dried root of *Rehmannia glutinosa Libosch*, is also called Shengdi. These drugs have been used singly or in compound drugs in water decoctions to fight hepatitis and other diseases for a long time in Chinese folk medicine [14–16], and modern pharmacological studies have confirmed their hepatoprotective effects [17,18]. It is encouraging that Tianjihuang (*Hypericum japonicum*) injections have been applied in a clinical setting and shown provide notable hepatoprotective effects [18]. With the deepening of this area of research, phytochemical analyses have also revealed that the major bioactive ingredients of *Hypericum japonicum* and *Salvia plebeian* are flavonoids [19,20], and that polysaccharides play a major role in the effects of *Radix Rehmanniae Recens* [21]. Meanwhile, many studies have suggested that both the *Hypericum japonicum* flavone (HJF) and the *Salvia plebeia* flavone (SPF) have antiviral and antioxidant effects [17,22–24]. Furthermore, it has also been shown that RRRP has an effect against skin oxidative stress in experimental mice [25].

In this work, we compare the anti-DHAV effects of an HRS prescription to the effects of its three individual ingredients *in vitro* and *in vivo*, and we confirm that HRS is a competitive candidate drug to use against DHAV infection. Moreover, the free radical scavenging activity of HRS was also studied to explore its hepatoprotective mechanisms.

## Materials and Methods

### Ethics statement

All animal experiments were carried out in accordance with the guidelines set forth by the Institutional Animal Care and Use Committee (IACUC) and Nanjing Agricultural University IACUC, and the protocol was approved by the IACUC with the project number 2012GGC15003. All efforts were made to minimize the number of animals used and their suffering. During the entire experimental session in vivo, the ducklings were infected with virus by intramuscular injection only once. The intramuscular injection was one of the most commonly methods used in animal and human without obvious pain, so we did not use analgesics. A humane endpoint was used during the study *in vivo*. To ameliorate suffering, the ducklings were humanely euthanized by CO_2_ gas when the typical clinical symptoms of DVH, such as opisthotonos and convulsions, appeared. CO_2_ euthanasia was an effective method to ameliorate suffering of the animals which can be used alone. So we didn’t use analgesics and anaesthetics in this process, either. The health status of the ducklings was monitored 6 times per day. During the entire monitoring time, there were no ducklings died as a result of opithotonos or convulsions. During the entire experimental session, the ducklings were carefully nursed to reduce all types of stress, and each process was carried out strictly in accordance with the regulations of the animal protection committee to minimize injuries.

### Reagents and virus

DMEM (GIBCO) supplemented with 100 IU/mL benzylpenicillin, 100 IU/mL streptomycin, glutamine (0.75 mg/mL) and 10% heat-inactivated fetal bovine serum was used as nutritive medium to cultivate the cells. For maintenance medium [26], which was used to dilute the drug and virus and to maintain the cells, the fetal bovine serum concentration was reduced to 1%. Dulbecco's Hanks balanced salt solution (D-Hank’s) was used to wash the duck embryo tissue and to block the cells. The reagent 3-(4, 5-dimethyl-2-thiazolyl)-2, 5-diphenyl tetrazolium bromide (MTT, Amresco Co.) was dissolved at 5 mg/mL in calcium and magnesium-free phosphate-buffered saline. Trypsin (Amresco Co.) was dissolved with D-Hank’s to a concentration of 0.25% and stored at -20°C. These reagents were filtered through two 0.22 μm millipore membranes, and the pH was adjusted to 7.4 with a solution of 5.6% NaHCO_3_. DMEM, MM and D-Hank’s were stored at 4°C, and MTT solution was stored in a brown bottle at 4°C. Ethanol, Dimethyl sulfoxide (DMSO, Lot no. 20141013) and other chemicals used in the experiments were analytical grade and were purchased from Guoyao Co., Ltd.

Superoxide dismutase (SOD) assay kit (Lot no. 20141211), catalase (CAT) assay kit (Lot no. 20141211), glutathione peroxidase (GSH-Px) assay kit (Lot no. 20141215), malondialdehyde (MDA) assay kit (Lot no. 20141211), nitric oxide synthase (NOS) assay kit (Lot no. 20141216) and total antioxidant capacity (T-AOC) assay kit (Lot no. 20141216) were purchased from Nanjing Jiancheng Bioengineering Institute.

The RNAiso Plus Reagent (Lot no. 9108), PrimeScript^™^ RT Master Mix Kit (Lot no. AK 3101) and SYBR^®^ Premix Ex Taq^™^ (Tli RNaseH Plus) Kit (Lot no. AK 5303) were Takara products.

DHAV (*LQ*_*2*_ strain) was provided by the Shandong Institute of Poultry. The TCID_50_ was 1 × 10^−4^ according to a Reed-Muench assay [27]. The analytic 50 TCID_50_ (5 × 10^−3^) was used for antiviral assays *in vitro*.

### Drug preparation

The *Radix Rehmanniae Recens* polysaccharide (RRRP) was extracted in our laboratory. The content of RRRP was 84.5% according to the phenol-sulfuric acid method [28]. The crude polysaccharide was purified by the Sevage method and by column chromatography using Sephadex G-200 and it didn’t contain flavone. The *Hypericum japonicum* flavone (HJF, net content of 60%) and *Salvia plebeia* flavone (SPF, net content of 70%) were purchased from Nanjing Zelang Medical Technology Co., Ltd. For HJF extract, its major flavones were quercetin and isoquercetin and it didn’t contain polysaccharide. As the reference component, the level of the quercetin was 10.35% according to HPLC analysis. For SPF extract, its major flavones were homoplantaginin, nepetin-7-glucoside and luteolin-7-glucoside and it didn’t contain polysaccharide. As the reference component, the level of the homoplantaginin in the extract was 9.59% according to HPLC analysis.

The proportion of each ingredient in the prescription of HRS was based on the formula theory of traditional Chinese medicine. The composition proportion of net HJF, SPF and RRRP in the prescription was 2:1:2. Based on our previous research, RRRP, HJF, SPF and HRS were diluted to their maximal safe concentrations of 1250 μg/mL, 12.500 μg/mL, 2000 μg/mL and 2500 μg/mL, respectively, in twofold serial dilutions with MM and then sterilized and stored at 4°C. For *in vivo* tests, the RRRP, HJF, SPF and HRS prescription was diluted to 5 mg/mL and stored at 4°C.

### Preparation of duck embryonic hepatocytes (DEHs)

DEHs were prepared as previously described in detail by Chen et al [6]. In brief, sterile livers were ground and digested to obtain single cells. Cells were then incubated in a humid atmosphere of 5% CO_2_ at 37°C at a seeding density that was adjusted to 0.8×10^6^−1.2×10^6^/mL. The hepatocytes grew into a monolayer and were taken as ready approximately 48 hours later.

### Comparison of anti-DHAV effects of the three single ingredients versus the combined prescription *in vitro* and *in vivo*

#### Comparison of antiviral activity *in vitro*

RRRP, HJF, SPF and HRS solutions were diluted to five concentrations from prepared concentrations. When DEHs had grown into monolayers in 96-well plates, after being cultivated for 48 hours, the wells in each 96-well plate were separated into three groups: cell control (CC) group, virus control (VC) group and drug group. A 100 μL volume of virus solution was added to each well except the CC wells, to which maintenance medium was added at an equal volume. The supernatant was removed and the cells were washed twice with D-Hank’s after the plates were incubated for 2 hours at 37°C in a humid atmosphere of 5% CO_2_. A volume of 100 μL RRRP, HJF, SPF and HRS solution in a series of concentrations was then added to the drug group wells. At the same time, 100 μL of maintenance medium was added to each well of the CC group and the VC group. All plates were then placed into a 5% CO_2_ incubator at 37°C. When clear cytopathic effects appeared in the VC group (after approximately 96 hours), the cells were washed twice with PBS, and then the MTT colorimetric method [29] was applied to measure the cytoactivity in each plate. The viral inhibitory rate was calculated based on the formula [30]: Viral inhibitory rate (%) = ($\overset{-}{A}\;$_drug + virus_−$\overset{-}{A\;}$_virus control_)/($\overset{-}{A\;}$_cell control_−$\overset{-}{A\;}$_virus control_) × 100%. Antiviral activity among RRRP, HJF, SPF and HRS was evaluated compared to the results of A_570_ and the viral inhibitory rate.

#### Comparison of clinical curative effect

A total of 210 one-day-old cherry valley ducklings (Purchased from the Chaoyang hatchery, Anhui province, China) that were not inoculated with a DHV vaccine were housed in wire cages (100 cm × 60 cm × 40 cm) in air-conditioned rooms at 37°C with 24 hours of light for the first several days. The temperature was then gradually decreased to room temperature and the light decreased to 12 hours per day. The ducklings were fed a commercial diet provided by the Feed Factory of the Jiangsu Academy of Agricultural Science. On the fourth day, the ducklings were challenged with 0.2 mL of DHAV by intramuscular injection, except for the ducks in the blank control (BC) group, and then randomly divided into RRRP, HJF, SPF, HRS and Virus Control (VC) groups. The ducklings in the separately reared BC group were injected with an equal volume of physiological saline. Two hours later, ducklings in the RRRP, HJF, SPF and HRS groups were administrated with prepared drug solutions at a dosage of 3 mg net drug per duckling by drinking water, once a day for five days. The dosage of drugs was determined based on our previous studies. All procedures involving animals were conducted strictly in accordance with the Chinese legislation for the use and care of laboratory animals, and the experimental use of animals and procedures were approved by the Nanjing Agricultural University Animal Care Committee. To ensure consistency across tests, ducklings in the BC and VC groups were treated with the same volume of solvent-added solution. To evaluate the clinical curative effect of each drug, the mortality rate and the time of death ending were recorded. Because DHAV-induced DVH is an acute disease, the ducklings died quickly once the typical clinical symptoms appeared. Therefore, to ameliorate suffering, the ducklings were humanely euthanized by CO_2_ gas when the typical clinical symptoms of DVH, such as opisthotonos and convulsions, appeared. Only when the pathological change was identified as DVH was the death counted. Dead ducklings were executed and disposed of in bio-safety containers in accordance with local standard protocols. The mortality rate in each group was calculated according to the formula: mortality rate (%) = the number of dead ducklings/the number in the sample group × 100%.

### Curative effect of HRS prescription and its hepatoprotective effect

#### Animal grouping and treatment

Based on results comparing the clinical effects of the HRS prescription to its single ingredients, the HRS prescription was indicated as the most effective drug against DVH. According to the feeding and management procedures described above, a total of 180 four-day-old cherry valley ducklings that were not inoculated with a DHV vaccine were divided into three groups: BC, VC and HRS. The following challenge and treatment procedures are the same as above. At the acute phase (4 hpi and 8 hpi) (hpi: hours post-injection [31]) and recovery phase (54 hpi), blood samples were randomly taken from 5 feathers collected from birds in each group, and these ducklings (15 feathers per group) were not included in the mortality rate.

#### Evaluation of hepatic injury

In the interest of evaluating the severity of DVH, both visual changes in pathological anatomy and the index of hepatic injury were studied. Once the typical clinical symptoms appeared, the ducklings were humanely euthanized by CO_2_ gas and the changes in pathological anatomy in their livers were graded and recorded. The degree was classified on a 0–5 point scale, with the degree increasing with increases in the aggravation of the hepatic injury. A 0 point score meant no pathological changes were found; 1 point: less than 10 hemorrhagic spots or only one ecchymosis appeared on the liver surface; 2 points: the color of the liver appeared essentially normal and the number of hemorrhagic spots was approximately 10 to 100; 3 points: the color of the liver slightly changed to red and one third of the liver surface appeared to have hemorrhagic spots; 4 points: the color of the liver slightly changed to yellowish red and half of the liver appeared to have hemorrhagic spots; and 5 points: the whole liver appeared to be experiencing diffuse hemorrhage, the color of the liver changed to yellowish red and more than 5 ecchymoses were identified on the liver surface. The average score was calculated with the following equation: (0 × the number of survival ducklings + 1 × the number of ducklings scored with 1 point + 2 × the number of ducklings scored with 2 points + …… + 5 × the number of ducklings scored with 5 points).

The plasma contents of alanine aminotransferase (ALT), aspartate aminotransferase (AST), albumin (ALB), lactate dehydrogenase (LDH), alkaline phosphatase (ALP) and total protein (TP) in the blood were tested at 4 hpi, 8 hpi and 54 hpi using an Automatic Biochemistry Analyzer (7180 Automatic Biochemistry Analyzer, HITACHI, Japan).

To investigate the free radical-scavenging activity of HRS *in vivo*, the plasma levels of SOD, CAT, GSH-Px, NOS, MDA and T-AOC were detected using the appropriate kit according to the kit instructions.

#### Relative DHAV gene expression in each group

Total RNA was extracted from blood samples with RNAiso Plus Reagent according to the kit instructions. Then, cDNA was synthetized using a PCR machine (2720 Thermal Cycler PCR instrument, Applied Biosystems, America) with a PrimeScript^™^ RT Master Mix Kit. The cycling program was 37°C for 15 min, 85°C for 5 s, and 4°C for 7 min. Finally, the semi-quantitative analysis of viral replication was conducted using a RT-PCR machine (StepOnePlus^™^ Real Time PCR instrument, Applied Biosystems, America) and a SYBR^®^ Premix Ex Taq^™^ (Tli RNaseH Plus) Kit. The primers for DHAV and β-actin were designed in our previous study [6]. The primer sequences used were as follows: DHAV forward: 5’- GCCACCCTTCCTGAGTTTGT-3’, DHAV reverse: 5’-TACCATTCCACTTCTCCTGCTT-3’; β-actin forward: 5’-CTTTCTTGGGTATGGAGTCCTG-3’, β-actin reverse: 5’-TGATTTTCATCGTGCTGGGT-3’. The reaction parameters were as follows: 95°C for 30 s, 95°C for 5 s (40 cycles) and 60°C for 30 s.

#### Correlation analysis

The correlation among hepatic function indexes, peroxidation damage evaluation indexes, mortality rate, viral gene expression levels and hepatic pathological severity scores at 54 hpi were determined by Pearson’s correlation coefficient using SPSS Software Package v.20.0.

### Statistical analysis

All data are expressed as the mean ± S.D. Duncan’s multiple range test was used to analyze the difference among groups and a χ^2^ -Test was used to analyze the difference in mortality rates with SPSS 20.0 software. The 2^-ΔΔCT^ method [32] was used to analyze relative gene expression data. Significant differences were considered as *p* < 0.05.

## Results

### Results from the comparison of anti-DHAV activity by the three single ingredients to the combined prescription, *in vitro*

The A_570_ values and viral inhibitory rates on DEHs are presented in Table 1. The A_570_ values of the HJF and HRS groups at different concentrations were significantly increased compared with the VC group (*p* < 0.05). However, only at a concentration of 1000 μg/mL was the A_570_ value of the SPF group significantly higher than that of the VC group (*p* < 0.05). The A_570_ value of the RRRP group was significantly higher than that of the VC group at a concentration of 78.125–312.500 μg/mL. The highest inhibitory rates of the HJF, SPF, RRRP and HRS groups were 37.0%, 20.2%, 32.6% and 103.9%, respectively. Furthermore, the viral inhibitory rate of the HRS group was generally higher than that of the HJF, SPF and RRRP groups, and the HJF group generally presented a higher viral inhibitory rate than the SPF and RRRP groups.

**Table 1:** The A_570_ values and viral inhibitory rates on DEHs.

Treatment	Concentration (μg/mL)	A_570_	Viral inhibitory rate (%)	Treatment	Concentration (μg/mL)	A_570_	Viral inhibitory rate (%)
HJF	12.500	0.537±0.005^c^	30.3	SPF	2000	0.273±0.002^cd^	6.6
	6.250	0.564±0.003^b^	34.6		1000	0.306±0.007^b^	20.2
	3.125	0.579±0.008^b^	37.0		500	0.289±0.007^bc^	13.2
	1.563	0.541±0.009^c^	30.9		250	0.279±0.005^cd^	9.1
	0.781	0.444±0.006^d^	15.4		125	0.279±0.007^cd^	9.1
CC		0.624±0.007^a^				0.500±0.011^a^	
VC		0.348±0.006^e^				0.257±0.009^c^	
Treatment	Concentration (μg/mL)	A_570_	Viral inhibitory rate (%)	Treatment	Concentration (μg/mL)	A_570_	Viral inhibitory rate (%)
RRRP	1250	0.449±0.009^d^	11.2	HRS	2500	0.352±0.003^b^	88.9
	625	0.448±0.015^cd^	10.9		1250	0.387±0.005^a^	103.9
	312.500	0.473±0.024^bc^	18.9		625	0.282±0.010^c^	59.0
	156.250	0.516±0.019^b^	32.6		312.500	0.200±0.008^d^	23.9
	78.125	0.478±0.023^bc^	20.5		156.250	0.219±0.004^d^	32.1
CC		0.727±0.010^a^		CC		0.378±0.009^a^	
VC		0.414±0.008^d^		VC		0.144±0.003^e^	

^a–e^ Data within a column without the same superscripts differ significantly (*p* < 0.05).

### Results showing anti-DVH effects in a comparison of the three single ingredients and the combined prescription

The mortality rates and the time of death ending in the HJF, RRRP, SPF and HJF groups are presented in Table 2. In the VC group, the mortality rate (74.3%) and mortality (26 birds) were both the highest. In contrast, in comparison to the VC group, the mortality (15 birds) and the mortality rate (42.9%) of the HRS group were significantly decreased (*p* < 0.05). The mortality rates in the HJF, SPF and RRRP groups were also reduced to 57.1%, 62.9% and 60.0%, respectively. The HJF group had a lower mortality rate and was not significantly different compared to the SPF and RRRP groups (*p* > 0.05). In the overall ranking of the time of death ending, the VC group was the longest lived (up to 144 hours), followed by the RRRP group (120 hours), the SPF group (120 hours), the HRS group (108 hours) and the HJF group (54 hours).

**Table 2:** Results of the three single ingredients and HRS prescription anti-DVH effects.

Group	Samples (feathers)	Final deaths (feathers)	Time of death ending (hpi)	Mortality rate (%)
HJF group	35	20	54	57.1^ab^
SPF group	35	22	120	62.9^ab^
RRRP group	35	21	120	60.0^ab^
HRS group	35	15	108	42.9^b^
VC group	35	26	144	74.3^a^
BC group	35	0	-*	0^c^

^a−c^ Data within a row without the same superscripts differ significantly (*p* < 0.05).

*Time of death ending was unable to record for no deaths appeared.

hpi: hours post-injection.

### Results showing the curative and hepatoprotective effects of HRS

#### Results showing the curative effect of HRS

**Fig 1:**
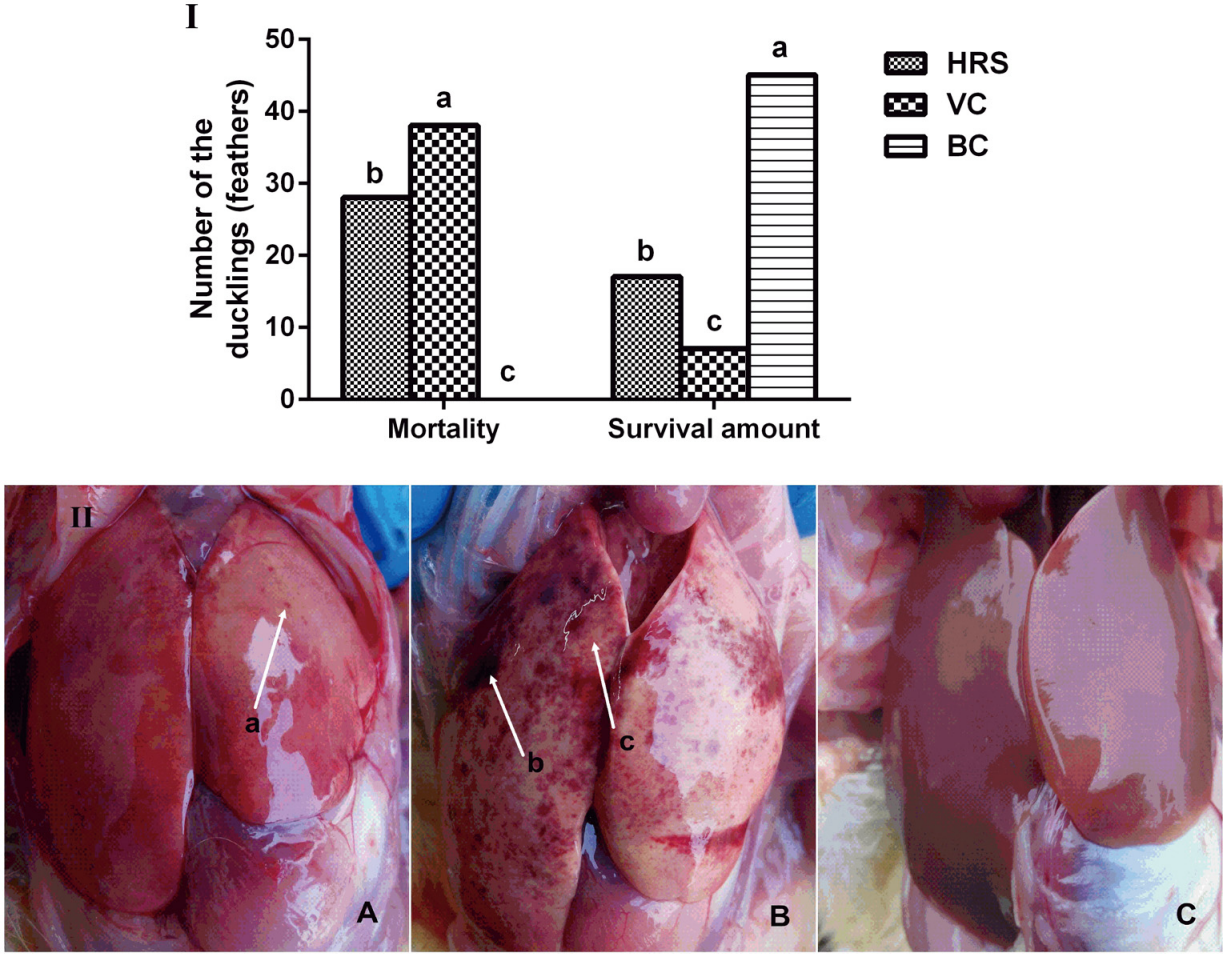
The curative effect of the HRS prescription and the Visual changes in pathological anatomy in each group. (I) The curative effect of the HRS prescription. ^a-c^ Bars in the same index without the same superscripts differ significantly (*p* < 0.05). (II) Visual changes in pathological anatomy in the HRS (A), VC (B) and BC (C) groups. Arrows a and c: hemorrhagic spots; arrow b: ecchymosis.

The curative effect of HRS on DVH is presented in Fig 1(I). The mortality rate in the VC group was the highest among the three groups and was significantly higher than that in the BC and HRS groups (*p* < 0.05). After treatment with HRS, the mortality rate in the VC group was decreased by 22.2%.

#### Visual changes in pathological anatomy and scores of hepatic pathological changes

Visual changes in pathological anatomy in the BC, VC and HRS groups are illustrated in Fig 1(II) (A, B and C). As picture C shows, the liver surface was lubricious and no change in pathological anatomy was observed in the BC group. In the VC group (picture B), a large number of hemorrhagic spots (arrow c) and ecchymoses (arrow b) were identified on the liver surface and the color of the liver changed to yellowish red. On the contrary, the pathological anatomy in the HRS group was clearly alleviated, in that they displayed fewer hemorrhagic spots (arrow a) and no ecchymosis were found on the liver surface in this group (picture A). Moreover, the color of the liver of the HRS group was much closer to that of the BC group than in the VC group.

The quantitative analysis of average liver injury severity in each group is illustrated in Table 3. The liver injury severity score in the BC group was zero, indicating no pathological change. However, the VC group presented the highest average injury severity score, which was significantly higher than that of the BC and HRS groups (*p* < 0.05).

**Table 3:** The number of ducklings corresponding to the different scores in each group.

Group	0 point (feathers)	1 points (feathers)	2 points (feathers)	3 points (feathers)	4 points (feathers)	5 points (feathers)	Average score (points)
HRS group	17	6	9	6	4	3	1.6^b^
VC group	7	3	5	8	12	10	3.0^a^
BC group	45	0	0	0	0	0	0^c^

^a-c^ Data within a row without the same superscripts differ significantly (*p* < 0.05).

#### Results of hepatic function biochemical indexes

Table 4 shows the hepatic function biochemical indexes in each group. All of these indexes are shown at 4 hpi, including the TP values at the three sampling times, which showed no significant difference among the BC, VC and HRS groups (*p* > 0.05).

**Table 4:** Results of hepatic function biochemical indexes in each group.

Index	Time (hpi)	HRS group	VC group	BC group
ALT (IU/L)	4	25.2±3.9^a^	27.0±3.3^a^	27.2±2.4^a^
	8	33.2±3.0^b^	43.0±2.8^a^	28.6±1.6^b^
	54	41.3±3.2^b^	67.4±3.7^a^	36.8±1.0^b^
AST (IU/L)	4	13.0±0.8^a^	14.0±2.1^a^	12.3±1.7^a^
	8	20.5±0.6^ab^	28.8±5.2^a^	14.0±1.0^b^
	54	16.7±4.7^ab^	21.2±2.1^a^	12.6±0.8^b^
TP (g/L)	4	24.7±1.1^a^	25.4±1.1^a^	24.7±0.7^a^
	8	27.8±1.0^a^	26.2±0.40^a^	28.0±1.2^a^
	54	25.8±1.1^a^	25.9±0.6^a^	28.1±0.5^a^
ALB (g/L)	4	10.3±0.5^a^	10.7±0.6^a^	10.9±0.5^a^
	8	11.3±0.3^a^	11.1±0.3^a^	12.3±0.6^a^
	54	11.4±0.4^ab^	10.8±0.1^b^	12.2±0.5^a^
LDH (IU/L)	4	379.8±18.6^a^	403.3±47.9^a^	375.8±54.5^a^
	8	474.0±10.6^b^	595.0±14.4^a^	307.8±31.0^c^
	54	1233.3±147.3^b^	4743.0±570.8^a^	312.2±13.7^c^

^a-c^ Data within a row without the same superscripts differ significantly (*p* < 0.05).

hpi: hours post-injection.

At 8 hpi, ALT, AST and LDH levels in the VC group were significantly higher than those in the BC group (*p* < 0.05). The levels of ALT and AST in the HRS group both decreased compared with levels in the VC group, but they were close to the levels in the BC group. The LDH level in the HRS group was significantly lower than that in the VC group. The levels of TP and ALB were almost the same among the BC, VC and HRS groups.

At 54 hpi, significant increases in the ALT, AST and LDH levels were detected in the VC group compared with levels in the BC group (*p* < 0.05). In the HRS group, the ALT and LDH levels were significantly lower than those in the VC group (*p* < 0.05), and the AST level decreased but was not significantly different compared to that in the VC group (*p* > 0.05). The levels of both ALT and AST in the HRS group were the same as in the BC group (*p* > 0.05).

#### Results of evaluation indexes of peroxidation damage

**Fig 2:**
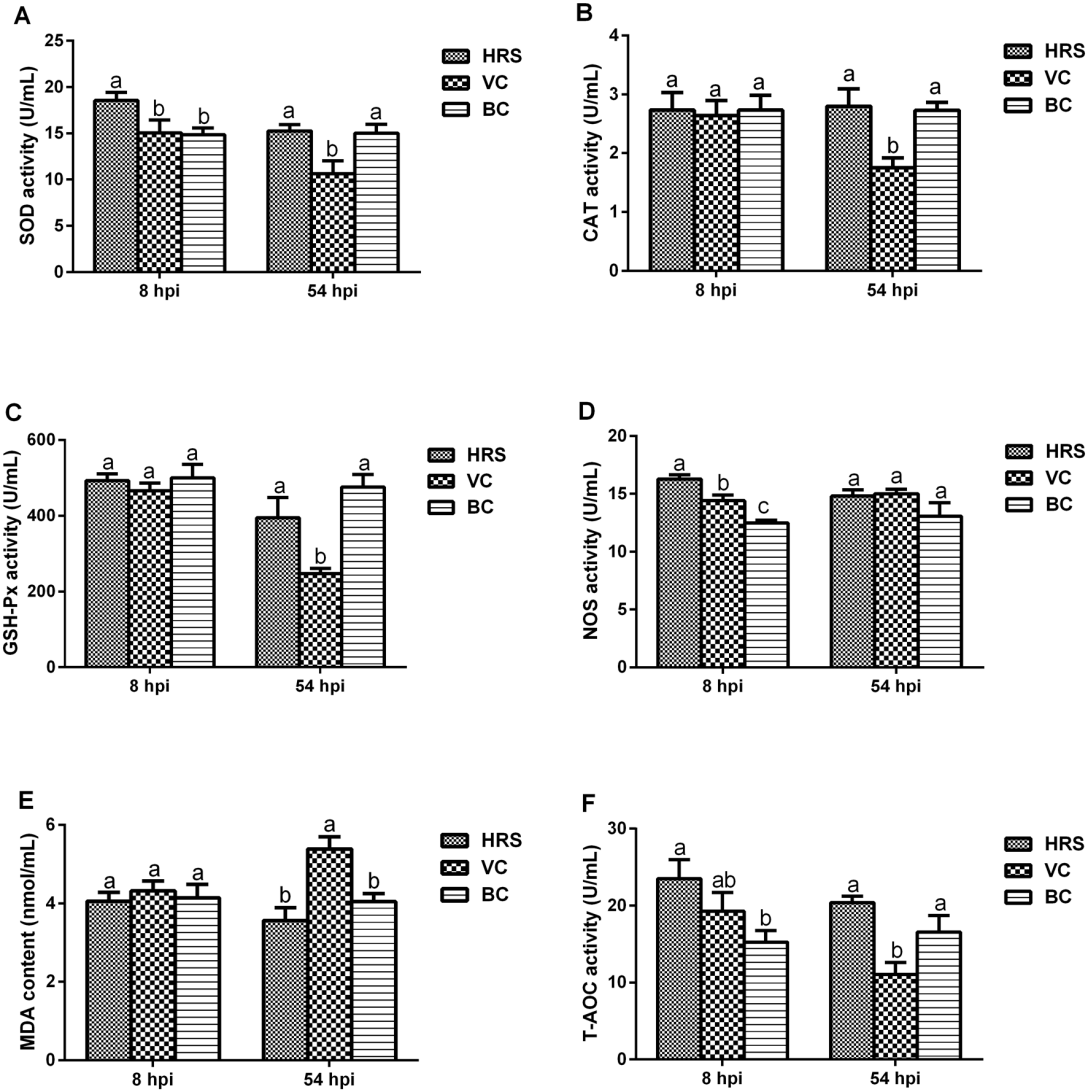
Peroxidation damage evaluation indexes at 8 hpi and 54 hpi (hpi: hours post-injection). ^a-c^ Bars in the same index at the same time point without the same superscripts differ significantly (*p* < 0.05).

The evaluation indexes of peroxidation damage are shown in Fig 2. The plasma levels of SOD, CAT, GSH-Px, MDA and T-AOC in the VC group were not distinct from those in the BC group; however, plasma NOS activity was significantly elevated in these animals at 8 hpi (*p* < 0.05). Furthermore, the plasma levels of CAT, GSH-Px, MDA and T-AOC were almost the same between the VC and HRS groups. In addition, the activity of both SOD and NOS was significantly increased in the HRS group compared with those in the VC group (*p* < 0.05).

At 54 hpi, the levels of plasma SOD, CAT, GSH-Px and T-AOC among the three groups showed the same relationship: the HRS group was close to the BC group, and both were significantly higher than the VC group (*p* < 0.05). The plasma MDA level in the HRS and BC groups was almost the same, and both were significantly lower than that of the VC group (*p* < 0.05). The plasma NOS level presented no difference among the three groups (*p* > 0.05).

#### Results of relative expression of DHAV in the blood

**Fig 3:**
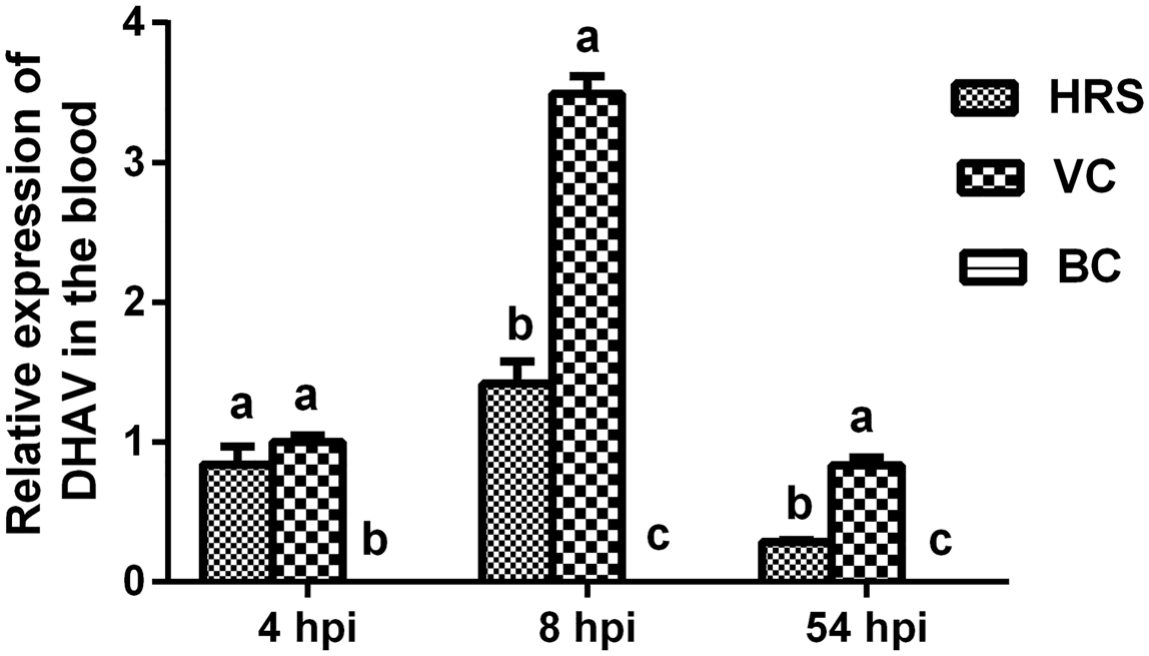
Relative DHAV gene expression levels in each group. Viral gene expression level in the VC group at 4 hpi was set to 1. ^a-c^ Bars at the same time point without the same superscripts differ significantly (*p* < 0.05). hpi: hours post-injection.

Relative gene expression of DHAV in the blood at 4 hpi, 8 hpi and 54 hpi is listed in Fig 3. Viral gene expression in the VC group at 4 hpi was set to 1.

The DHAV gene expression level in the VC and HRS groups was significantly higher than that of the BC group at all time points (*p* < 0.05). At 8 hpi and 54 hpi, the viral gene expression level in the VC group was 1.46 times and 1.93 times higher, respectively, and was significantly higher than that in the HRS group (*p* < 0.05). The rate of viral gene expression inhibition by HRS was as high as 59.3% at 8 hpi and 65.9% at 54 hpi. [The viral gene expression inhibition rate was calculated using the formula: Viral gene expression inhibitory rate (%) (at the same time point) = (the relative viral gene expression in the VC group—the relative viral gene expression in the drug group)/the relative viral gene expression in the VC group × 100%].

#### Results of the correlation analysis

The Pearson correlation coefficients among hepatic function indexes, evaluation indexes of peroxidation damage, mortality rate, viral gene expression level and hepatic pathological severity score at 54 hpi are listed in Table 5. As shown in this list, ALT, AST and LDH were positively correlated with MDA, NOS and viral gene expression level and mortality rate but negatively correlated with SOD, CAT, GSH-Px and T-AOC. The correlation analysis also showed that ALB was positively correlated with T-AOC but negatively correlated with SOD, CAT, NOS, GSH-Px, MDA, viral gene expression level and mortality rate. Moreover, the hepatic pathological severity score was significantly and positively correlated with the mortality rate and viral gene expression (*p* < 0.01).

**Table 5:** The Pearson correlation coefficients among hepatic function indexes, peroxidation damage evaluation indexes, mortality rate, viral gene expression level and hepatic pathological severity score at 54hpi.

	ALT	AST	LDH	ALB	hepatic pathological severity score
SOD	-0.983**	-0.855*	-0.970**	-0.794	0.010
CAT	-0.981**	-0.849*	-0.967**	-0.787	0.021
GSH-Px	-0.976**	-0.990**	-0.988**	-0.969**	-0.385
NOS	0.681	0.896*	0.725	-0.939**	0.839
MDA	0.924**	0.730	0.899*	-0.651	-0.216
T-AOC	-0.849*	-0.608	-0.815	0.518	0.373
Viral gene expression level	0.979**	0.988**	0.990**	-0.965**	0.976**
Mortality rate	0.394	0.693	0.450	-0.767	0.974**

* p < 0.05.

** *p* < 0.01.

hpi: hours post-injection.

## Discussion

The MTT method is one of the most convenient assays for testing cellular activity in cell cultures, *in vitro* [33]. As an intuitive index used to evaluate the activity of cells, the higher the A_570_ value, the higher the activity and the better the antiviral activity displayed by the drug [34]. The A_570_ values of the HJF, SPF, RRRP and HRS groups in *in vitro* experiments were significantly higher than those in the corresponding VC group at 0.781–12.500 μg/mL, 1000 μg/mL, 78.125–312.500 μg/mL and 156.250–2500 μg/mL (Table 1) (*p* < 0.05). These results indicate that all of these drugs have significant anti-DHAV effects *in vitro*. The viral inhibition rate as an index directly reflects the antiviral intensity of the drug [35]. In comparison to the highest viral inhibition rate of the four drug groups (HJF: 37.0%, SPF: 20.2%, RRRP: 32.6%, and HRS: 103.9%), the HRS group presented the best result, and therefore had better and stronger anti-DHAV activity *in vitro* than the three single ingredients (Table 1). To further evaluate the anti-DHAV effects of the three single ingredients and the combined HRS prescription, we tested the curative effect of these drugs in artificially infected ducklings. The mortality rate is the most useful index for evaluating the clinical curative effect. Although the mortality rates in groups given one of the three single ingredients or the HRS prescription groups were lower than in the VC group, only the HRS group showed a significant decrease (*p* < 0.05) (Table 2). This result was consistent with our *in vitro* results (Table 1) and demonstrated that the HRS prescription conferred a more superior clinical curative effect than any other three single drugs. Therefore, further research is required to determine the anti-DHAV mechanisms that function in the HRS prescription.

In this verification experiment, the results from the final mortality rate experiment (Fig 1) were consistent with those in the preliminary experiment (Table 2). The visual changes in pathological anatomy were clearly alleviated after treatment with HRS (Fig 1). The hepatic pathological severity score provided a quantitative analysis index of the liver injury severity: the higher the score, the more serious the injury. The score in the VC group was the highest and was significantly higher than that in the HRS group (*p* < 0.05) (Table 3), which was consistent with the visual changes observed in pathological anatomy (Fig 1). These results serve as evidence that validates the effective clinical curative effect of this HRS prescription for treating DVH. Moreover, the significantly positive correlation between hepatic pathological severity scores and mortality rates further confirms that the curative effect of HRS against DHV was closely related to its actions that alleviated hepatic injury (Table 5).

The biochemical markers AST, ALT, TP, ALB and LDH are widely used to diagnose many types of human hepatitis [26,36,37]. These markers are of great significance in differential diagnoses, curative effect monitoring and evaluations of hepatitis. In this experiment, all of the biochemical markers that were tested showed no significant difference among the three groups at 4 hpi (Table 4). However, in the VC group, the levels of ALT, AST and LDH were significantly higher than in the BC group at 8 hpi (*p* < 0.05). This indicates that the hepatic injury appeared at 4 to 8 hours following infection with DHAV and that the biochemical markers AST, ALT and LDH displayed higher sensitivity than the other markers. The decrease in the three markers in the HRS group suggests that HRS effectively protects the liver from injury caused by DHAV infection during the acute phase. Similarly, the AST, ALT and LDH levels in VC group remained the highest among the three groups at 54 hpi. Meanwhile, the level of ALB was significantly lower than that in the BC group. In the HRS group, the levels of AST, ALT and ALB were the same as those in the BC group at 54 hpi. This indicates that the hepatic injury remained serious and that the prognosis of the disease was poor in the VC group ducklings. Conversely, HRS alleviated the injury and had a protective and curative effect during the recovery phase. The correlation analysis also showed that hepatic function indexes (AST, ALT, and LDH) were positively correlated with the mortality rate (Table 5). These results suggest that hepatic injury was positively correlated with the mortality rate. These results therefore confirm that HRS confers hepatoprotective effect from another angle.

Health status and viral gene expression levels are closely related, and a higher viral gene expression level indicates a worse health status [38]. The replication cycle of DHAV includes adsorption, penetration, uncoating, biosynthesis, assembly and release, and requires approximately 6 to 8 hours. In the early period, before 8 hpi, many specific defense mechanisms in the body have not yet been triggered, and the antiviral effect of a drug mainly depends on its direct inactivating activity [8]. Although DHAV gene expression levels in the blood in the VC group was higher than that in the HRS group, there was no significant difference between them at 4 hpi (*p* > 0.05) (Fig 3). This indicates that HRS possesses a faint inhibitory effect against viral proliferation at this time point. After being challenged with DHAV for 8 hours, the blood DHAV gene expression level increased significantly in both the VC and HRS groups, and the level in the VC group was significantly higher than that in the HRS group (*p* < 0.05). This suggests that the virus finished at least one replication cycle and that a large amount of virus had been released into the blood. HRS presented an effective ability to inhibit the proliferation of DHAV *in vivo* (Fig 3), which was consistent with the experiment *in vitro* (Table 1). In the recovery phase (54 hpi), the DHAV gene expression levels in the VC and HRS groups were clearly decreased, and the expression level in the HRS group was much lower (Fig 3). This reveals that DHAV was going through the natural process of being gradually cleaned out after peak virus replication. Meanwhile, the correlation analysis results also showed that DHAV gene expression levels were positively correlated with hepatic injury indexes and the pathological severity score (Table 5). This indicates that when the viral load in the blood increases, more hepatocytes are infected, leading to exacerbation of the hepatic injury. Hence, it is possible that HRS exerts its hepatoprotective effect by inhibiting the proliferation of DHAV.

Oxidative stress is one of the negative effects produced by free radicals in the body, and it is believed to be an important factor leading to aging and disease [39]. A large amount of evidences accumulated over the past decades suggested that patients with RNA virus infections are under oxidative stress [5]. In response to this damage, cells protect themselves by using a variety of free radical scavengers, such as SOD, CAT, GSH-Px, and vitamins E and C [40]. T-AOC level is an important and direct index used to evaluate total radical scavenging activity. NO is a relatively short-lived inorganic free radical that is synthesized by the different isoforms of nitric oxide synthase NOS (i.e., nNOS, eNOS and iNOS) and can cause oxidative stress and fight against bacteria, parasites and viruses [41]. MDA is one of the main products of lipid peroxidation, and its elevated level reflects the degree of lipid peroxidation injury [42].

The NOS plasma levels in both the VC and HRS groups were significantly higher than that in the BC group at 8 hpi (*p* < 0.05), and the level in the HRS group was the highest (Fig 2). Meanwhile, the SOD and T-AOC plasma levels were also significantly increased (*p* < 0.05). This indicates that NO might play a pivotal role against viral infections, and that HRS enhances its synthesis to more effectively inhibit viral proliferation. However, all the other indexes in the VC group were at the same level as those in the BC group, which might indicate that the metabolism of free radicals was in an almost balanced state, despite the injury (Table 4). In addition, the elevated levels of plasma SOD and T-AOC suggested that HRS enhanced the free radical scavenging effect. In the recovery phase (54 hpi), all of the indexes were significantly different between the VC and BC groups except NOS (*p* < 0.05). This suggests that the balance in free radical metabolism had been destroyed, resulting in large amounts of free radicals accumulating in the body. Meanwhile, the correlation analysis results showed that the hepatic function indexes, including ALT, AST and LDH, were positively related to MDA and NOS and negatively related to SOD, CAT, GSH-Px, and T-AOC (Table 5). This indicates that the large number of free radicals being generated caused the hepatic injury to become aggravated, resulting in significant increases in the levels of ALT, AST and LDH in the VC group (Table 4). Therefore, it is clear that peroxidation damage is an important contributor to DHAV-induced hepatic injury. The level of NOS in the VC group seemed to bounce back to a normal level, but a large number of other types of free radicals remained, except for NO and T-AOC, which was significantly decreased, suggesting that the free radical scavenging effect was attenuated (Table 5). It therefore made no sense to change the imbalance. On the contrary, in ducklings treated with HRS, all of the indexes presented as no difference than those in the BC group, suggesting that the hepatoprotective effect of HRS might be related to its antioxidant activity. However, there were dead ducklings after the balance returned and this remains unexplained. Further investigated is therefore required to further clarify these mechanisms.

## Conclusion

The HRS prescription had superior anti-DHAV activity compared to the three single drugs both *in vivo* and *in vitro*. Research into the curative mechanism suggests that HRS confers a hepatoprotective effect that is closely linked to its antioxidant activity. These results indicate that the HRS prescription can be expected to be developed into a new candidate of anti-DHAV drug.
